# PRCC reduces the sensitivity of cancer cells to DNA damage by inhibiting JNK and ATM/ATR pathways and results in a poor prognosis in hepatocellular carcinoma

**DOI:** 10.1186/s13578-021-00699-x

**Published:** 2021-10-29

**Authors:** Chunying Liu, Xuejing Lin, Bin Sun, Ziming Mao, Lei Chen, Haihua Qian, Changqing Su

**Affiliations:** 1 Department of Molecular Oncology, Eastern Hepatobiliary Surgery Hospital, Navy Military Medical University, Shanghai, 200438 People’s Republic of China; 2 National Center for Liver Cancer, Navy Military Medical University, Shanghai, 201805 People’s Republic of China; 3 Department of Endocrinology, Shanghai Ninth People’s Hospital, Shanghai Jiao-Tong University School of Medicine, Shanghai, 200011 People’s Republic of China

**Keywords:** DNA damage, Hepatocellular carcinoma, JNK signaling pathway, PRCC, Prognosis

## Abstract

**Background and aim:**

The proline rich mitotic checkpoint control factor (PRCC) is involved in the splicing process of pre-mRNA. This study aims to elucidate PRCC molecular function, regulatory mechanism and diagnostic value in hepatocellular carcinoma (HCC).

**Methods:**

The tissue microarray and serum samples from HCC patients were used to investigate the clinical value of PRCC. The biological function and molecular mechanism of PRCC were demonstrated by cell biology, biochemical and animal experiments. The relationship between PRCC and intratumoral heterogeneity (ITH) was analyzed by bioinformatics.

**Results:**

PRCC was highly expressed in HCC tissues and related to the poor prognosis of HCC patients, its contents were elevated in the preoperative sera of HCC patients. PRCC exhibited high application potential as a substitute or adjuvant of alpha-fetoprotein (AFP) for clinical diagnosis of HCC. It had no significant effect on the proliferation of cancer cells, but could inhibit spheroid formation and metastasis of HCC cells in vitro and in vivo. The high ectopic expression of PRCC made cancer cells insensitive to DNA damage, and enhanced the heterogeneity of HCC cells by inhibiting the JNK/ATM/ATR/ATF2 axis. The HCC patients with high PRCC expression had high ITH, which corresponded to a short overall survival in patients.

**Conclusions:**

PRCC has high application potential as a substitute or adjuvant of AFP for clinical diagnosis of HCC. The high ectopic expression of PRCC not only caused HCC cells to resist to cell death induced by DNA damage, but also endowed cancer cells with numerous DNA mutations to become increasingly heterogeneous, finally leading to a poor prognosis in HCC patients. These data suggested PRCC could be a promising therapeutic target in HCC patients.

**Supplementary Information:**

The online version contains supplementary material available at 10.1186/s13578-021-00699-x.

## Introduction

Hepatocellular carcinoma (HCC) is currently one of the most common malignant tumors, its morbidity and mortality have remained high for a long time [1]. Metastasis and recurrence after surgery, insensitivity to radiotherapy and chemotherapy, and high heterogeneity of HCC have become the bottleneck to improve the long-term efficacy after treatment [2, 3]. The sensitivity and specificity of existing diagnostic markers and therapeutic targets for HCC do not meet the clinical requirements [4]. Therefore, further research in molecular mechanisms of HCC occurrence and development is of great significance for clinical diagnosis and treatment of HCC.

PRCC (Gene ID: 5546) is a protein element involved in the pre-mRNA splicing process. The fusion protein formed by PRCC and transcription factor binding to IGHM enhancer 3 (TFE3) increases the activity of TFE3 and promotes the transcription of serpin family E member 1 (*SERPINE1*) [5]. The recruitment of phosphorylated PRCC to precursor mRNA activates the human spliceosome B complex [6]. PRCC participates in the formation of spliceosome [7, 8]. In addition, PRCC is involved in checkpoint control, colony formation, cell apoptosis, and can interact with mitotic arrest deficient 2-like protein 2 (MAD2B) and transfer it into nucleus to perform function [9].

There is no research on the role and mechanism of PRCC in HCC up to now. This study found that PRCC was highly expressed in HCC cells and tissues, and its contents were elevated in the preoperative sera of most HCC patients. Research on the clinical diagnostic significance of PRCC and its molecular mechanism in HCC will help to find new diagnostic indicators and treatment targets for HCC.

## Materials and methods

### Sample collection and PRCC detection

This study was approved by the Committee on Ethics of Medicine, Navy Military Medical University (Shanghai, China). All patients signed informed consents. Sixty-six pairs of human HCC and adjacent tissue samples were collected and made into HCC tissue microarray. PRCC expression in HCC and adjacent tissues was detected by immunohistochemistry (IHC). AlgorithmS program of ImageScope software (Aperio) was used to calculate the histochemical score. The median of the scores was selected to distinguish between high and low levels of PRCC. The correlations between PRCC and clinicopathological characteristics and survival were analyzed.

The preoperative serum samples from 105 HCC patients and 24 healthy volunteers were collected and stored at −80 ℃. The enzyme-linked immunosorbent assay (ELISA) detection kit (YX-H86033, Shanghai Sinobestbio Biotechnology Co., Ltd, Shanghai, China) was used to detect the concentration of PRCC in sera. All procedures were followed the kit instructions. ROC (receiver operating characteristics) analysis was performed to determine whether the concentration of PRCC in sera can be used as a diagnostic index for HCC.

### Cell lines and cell culture

The human HCC cell lines, including Hep3B, Huh7, QGY-7701, LM3 and immortalized liver cells WRL-68, were all provided by the Chinese Academy of Sciences Stem Cell Bank. The cells were cultured in DMEM with 10% FBS (Gibco, USA) and incubated in a CO_2_ incubator at 37 °C.

### Overexpression and knockdown of *PRCC*

The coding sequence (CDS) of PRCC (NM_005973.5) was synthesized and constructed into pLV-EF1α-EGFP-N plasmid (Inovogen, VL3311) to generate the *PRCC* overexpression vector. The short hairpin RNA 1 (shRNA1) and shRNA2 sequences were designed and constructed into pLKO.1 plasmid (Addgene, Plasmid #10878) to generate the *PRCC* knockdown vectors. The shRNA1 and shRNA2 sequences were as follows: 5’-ccgggctggtgcttattatcaggatctcgagatcctgataataagcaccagctttttg-3’ (shRNA1); 5’-ccggacaccagatcacatatcttatctcgagataagatatgt gatctggtgttttttg-3’ (shRNA2). The overexpression and knockdown vectors were packaged into lentiviruses which were used to infect the target cells. The cell sublines with overexpression or knockdown of *PRCC* were confirmed by Western Blot.

### Quantitative reverse transcription PCR (RT-qPCR)

Total RNA was extracted with Trizol reagent (Invitrogen, USA), and then the transcriptional levels of genes were tested by RT-qPCR. The primers involved were shown in Additional file 1: Table S1.

### Animal experiment

Animal experiments were approved by the Committee on Ethics of Medicine, Navy Military Medical University. All animals received humane care according to the criteria outlined in the “Guide for the Care and Use of Laboratory Animals.” Eighteen purebred 6-weeks-old Balb/c male nude mice were divided into three groups: blank control group (Hep3B), negative control group (Hep3B-EGFP) and PRCC overexpression group (Hep3B-PRCC OE). Each group contained 6 mice, and the corresponding cell sublines were injected into the tail vein (8 × 10^5^ cells/150 μl per mouse). Thirty days later, mice were sacrificed painlessly, and dissected to observe the tumor formation in lungs, livers and kidneys. HE staining was performed.

### Spheroid assay and immunofluorescence (IF)

One thousand cells per 150 μl (diluted in DMEM) were mixed with 150 μl Matrigel and spread evenly in a 24-well plate. After cultured for one week, the cell spheres appeared. The cell spheres were collected and the immunofluorescence procedure was carried out. The antibodies used were shown in Additional file 1: Table S2.

### Inhibition of cell proliferation and cell cycle

After incubated for 24 h in 96-well plates, cells were treated with ultraviolet (UV) irradiation with a dose of 5 J/m^2^ [Dose (mJ/cm^2^) ^=^ Exposure time (s) × Output intensity (mW/cm^2^)] and cultured for another 24 h, or cells were treated with 30 μM Cisplatin (DDP) for 24 h. Then CCK8 assay was carried out, and the inhibition rate was calculated.

After cultured for 24 h in petri dishes, cells were treated with UV irradiation with a dose of 5 J/m^2^ and cultured for another 24 h, or cells were treated with 30 μM DDP for 24 h. The cells were collected and the flow cytometry was used for cell cycle detection.

### Western blot

The cells were collected and lysed with RIPA lysis buffer (1% PMSF, 1% phosphatase inhibitor and 1% protease inhibitor were added). The Western blot procedure was carried out as usual. The antibodies involved were shown in Additional file 1: Table S2.

### Analysis of intratumoral heterogeneity (ITH)

The single nucleotide variation (SNV) files based on whole-exome sequencing (WES) of 365 patients with HCC were downloaded from the Cancer Genome Atlas (TCGA)—liver and intrahepatic bile ducts (LIHC) project of the website (https://portal.gdc.cancer.gov/). Mutant-allele tumor heterogeneity (MATH) is a simple, quantitative, and universally applicable method for assessing ITH. R program was used to calculate MATH [10]. The calculation program was described in the Additional file 1.

Based on the results of RNA Seq V2 RSEM in TCGA database of liver cancer, the samples were divided into high and low expression groups of PRCC. Then the differences of ITH between the two groups were analyzed. The relationship between ITH and overall survival was analyzed with follow-up data.

### Statistical analysis

Graphpad Prism 5 and SPSS 19.0 software were used for statistical analysis. Data were expressed as‾x ± SD (standard deviation). Analysis of Variance (ANOVA) was used for comparison between groups. The gray value was analyzed by Image-Pro Plus 6.0 software. The statistical graphs were made with Graphpad Prism 5 and Adobe Illustrator CS5 software. *P* < 0.05 indicated that the difference was statistically significant.

## Results

### PRCC is upregulated in HCC tissues and sera of patients and associated with poor prognosis

**Fig. 1:**
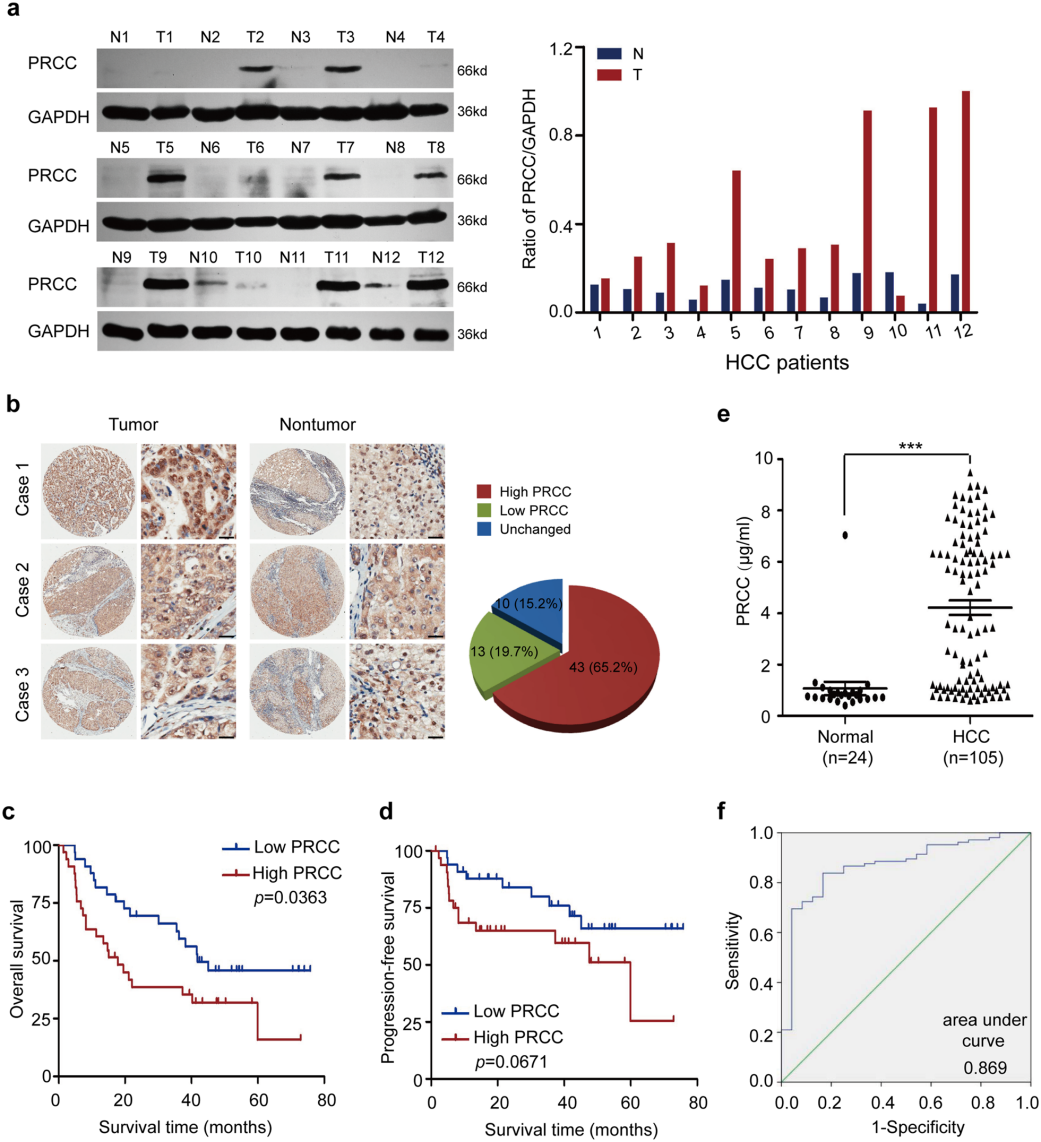
PRCC protein was highly expressed in HCC and was related to poor prognosis of patients. **a** PRCC expression in HCC and adjacent normal tissues examined by Western blot (N, normal; T, tumor). GAPDH was a loading control. **b** PRCC expression in HCC tissue microarray examined by IHC. Bar = 50 μm. **c**, **d** Overall survival and progression-free survival analysis of PRCC in HCC patients. **e** PRCC levels in sera of 105 HCC patients and 24 healthy volunteers examined by ELISA (***, *P* < 0.001). **f** ROC analysis of PRCC as a diagnostic index for HCC

The expression of PRCC in 12 pairs of HCC and adjacent normal tissues was detected by Western blot. The results showed that the protein level of PRCC in HCC tissues was significantly higher than that in adjacent normal tissues in 10 pairs of clinical samples, and was slightly lower in only one pair of samples, the remaining 1 pair of HCC tissue and adjacent tissue were equivalent expression for PRCC (Fig. 1a). IHC was performed on HCC tissue microarray including clinical samples from 66 HCC patients, and found that PRCC expression was elevated in HCC tissues compared with the adjacent tissues in 43 (65.15%) pairs of samples, and was reduced in 13 (19.70%) pairs of samples, there was no significant difference in its expression in the remaining 10 (15.15%) pairs of samples (Fig. 1b). ImageScope software (Aperio) was used to score the expression of PRCC in HCC tissues, and the median value of the scores was used to distinguish the groups with high and low levels of PRCC. Combined with analysis of the patients' clinicopathological characteristics and follow-up data, it was found that PRCC was significantly related to AFP level, portal vein tumor thrombus (PVTT) and TNM stage (Table 1). The results of survival analysis showed that the overall survival (OS) of HCC patients with high level of PRCC was significantly shorter than that of patients with low level of PRCC. The protein level of PRCC had no significant correlation with the progression-free survival (PFS) (Fig. 1c, d). The result was consistent with the analysis of the TCGA database (Additional file 1: Fig. S1).

**Table 1:** Correlation of PRCC expression and clinical features of HCC patients

Variables	Low (n = 33)	High (n = 33)	*P*-value
Gender, n (%)			0.741
Female	6 (18.2)	5 (15.2)	
Male	27 (81.8)	28 (84.8)	
Age, n (%)			0.218
≤ 50	14 (42.4)	19 (57.6)	
> 50	19 (57.6)	14 (42.4)	
HBV, n (%)			0.689
Negative	4 (12.1)	3 (9.1)	
Positive	29 (87.9)	30 (90.9)	
Cirrhosis, n (%)			0.689
No	4 (12.1)	3 (9.1)	
Yes	29 (87.9)	30 (90.9)	
AFP, n (%)			0.032
Negative, < 20 µg/L	14 (42.4)	6 (18.2)	
Positive, ≥ 20 µg/L	19 (57.6)	27 (81.8)	
Tumor size, n (%)			0.398
≤ 3 cm	10 (30.3)	7 (21.2)	
> 3 cm	23 (69.7)	26 (78.8)	
Tumor number, n (%)			0.492
= 1	6 (18.2)	4 (12.1)	
> 1	27 (81.8)	29 (87.9)	
PVTT, n (%)			0.046
No	6 (18.2)	1(3.0)	
Yes	27 (81.8)	32 (97.0)	
TNM stage, n (%)			0.030
I and II	10 (30.3)	3 (9.1)	
III and IV	23 (69.7)	30 (90.9)	

In addition, ELISA was used to detect PRCC concentration in sera of 105 HCC patients and 24 healthy volunteers. The results showed that PRCC concentration in sera of HCC patients was significantly higher than that of healthy volunteers, and the difference was statistically significant (*P* < 0.001) (Fig. 1e). Combined with the clinical diagnosis results, ROC analysis was performed through SPSS 19.0 software. The cutoff value of PRCC concentration in sera was 0.99 μg/ml, which meant that the patient would be more likely to develop HCC if his cutoff value was higher than 0.99 μg/ml. The area under the ROC curve (AUC) of PRCC was 0.869 (*P* < 0.05). The sensitivity of PRCC as a diagnostic index for HCC was 84%, and the specificity was 83% (Fig. 1f). Compared with AFP (based on randomly collected clinical data, the sensitivity of AFP for diagnosis of HCC was 59.05%, 62/105), PRCC had significant advantages in the sensitivity for HCC diagnosis. Therefore, PRCC was a potential substitute or adjuvant of AFP for clinical diagnosis of HCC. The above results indicated that the protein level of PRCC was generally high in HCC, which was related to a poor prognosis of HCC patients. It could be detected in sera and had high sensitivity as a diagnostic index for HCC.

### PRCC has no significant effect on the proliferation of HCC cells

**Fig. 2:**
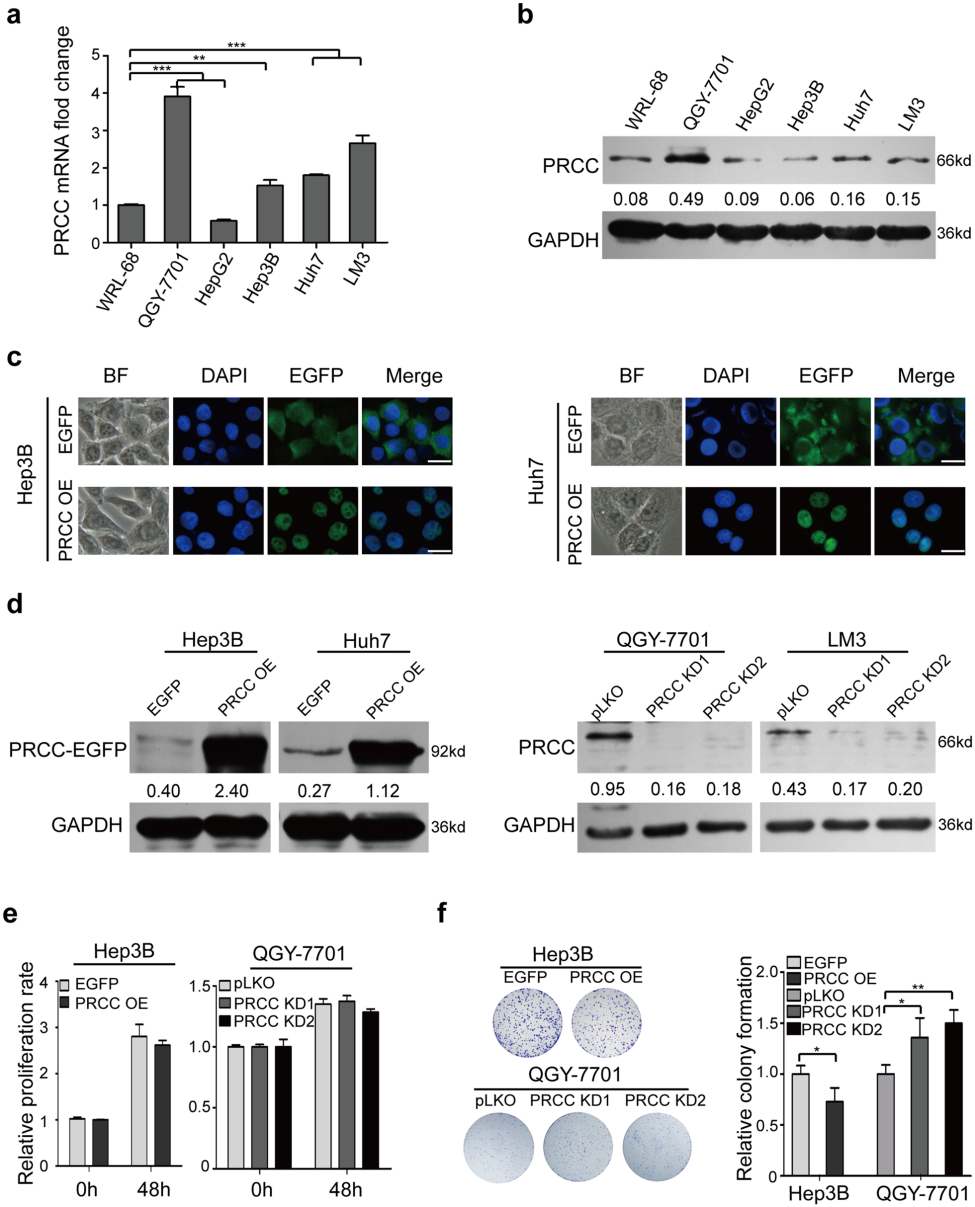
PRCC had no significant effect on the proliferation of HCC cells. **a** *PRCC* was detected by RT-qPCR in different HCC cell lines (**, *P* < 0.01; ***, *P* < 0.001). **b** PRCC was detected by Western blot in HCC cell lines. The numbers below the bands were the ratios of the gray values of PRCC normalized to that of GAPDH. **c** Fluorescence microscopy showed that PRCC located in nuclei. Bar = 25 μm. **d** PRCC was detected by Western blot in the PRCC OE and KD cell sublines. The numbers below the bands were the ratios of the gray values of PRCC to that of GAPDH. **e** CCK8 assay showed that PRCC had no significant effect on the proliferation of HCC cells. **f** Colony formation assay showed that PRCC inhibited the colony forming ability of HCC cells. (*, *P* < 0.05; **, *P* < 0.01)*.* All data were representative of three independent experiments

The mRNA and protein levels of PRCC in HCC cell lines were detected by RT-qPCR and Western blot, respectively. The results showed that the expression of PRCC in HCC cell lines (except HepG2) was generally higher than that in immortalized liver cells WRL-68 (Fig. 2a, b). Because HepG2 cells grew in colony state and were inconvenient for morphological observation, they were not selected as experimental cells. Instead, Hep3B and Huh7 cells with relatively low PRCC expression were selected to prepare the overexpressed cell sublines. QGY-7701 and LM3 cells with relatively high PRCC expression were selected to prepare the knockdown cell sublines. The exogenous PRCC-EGFP was stably expressed in the Hep3B (Hep3B-PRCC OE) and Huh7 (Huh7-PRCC OE) cell sublines. The transcriptional expression of *PRCC* was stably knocked down by two different shRNA vectors in the QGY-7701 (QGY-7701-PRCC KD) and LM3 (LM3-PRCC KD) cell sublines. Fluorescence microscopy showed that PRCC located in cell nuclei (Fig. 2c). The overexpression and knockdown of PRCC in cells was also confirmed by Western blot (Fig. 2d). The results of cell counting kit-8 (CCK8) assay showed that PRCC had no significant effect on the proliferation of HCC cells (Fig. 2e and Additional file 1: Fig. S2a). However, the results of colony formation assay showed that PRCC could inhibit the colony forming ability of HCC cells (Fig. 2f and Additional file 1: Fig. S2b).

### PRCC suppresses HCC cell spheroid formation

**Fig. 3:**
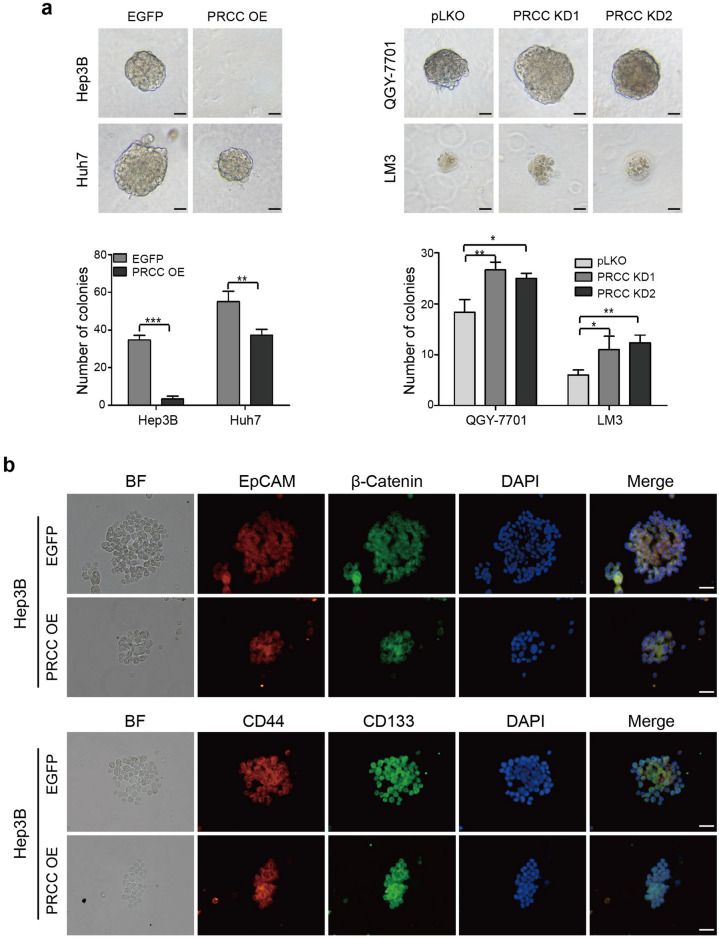
PRCC suppressed HCC cell spheroid formation. **a** Three-dimensional spheroid assay was used to detect the spheroidizing ability of HCC cells (*, *P* < 0.05; **, *P* < 0.01; ***, *P* < 0.001). Bar = 50 μm. **b** Immunofluorescence of stem cell markers including EpCAM, β-catenin, CD44 and CD133. Bar = 40 μm. All data were representative of three independent experiments

A three-dimensional spheroid assay was used to detect the spheroidizing ability of HCC cells. In the PRCC overexpression group, the ability of cancer cells to form spheroids was inhibited. Knockdown of PRCC promoted the formation of spheroids (Fig. 3a). The stem cell markers in Hep3B cell spheres were observed by immunofluorescence staining, and it was found that the four stem cell markers including EpCAM, β-catenin, CD44 and CD133 were all positive (Fig. 3b), demonstrating that the spheroid cells had the characteristics of stem cells. In summary, the high expression of PRCC had an inhibitory effect on cell spheroid formation.

### PRCC inhibits HCC cell metastasis in vitro and in vivo

**Fig. 4:**
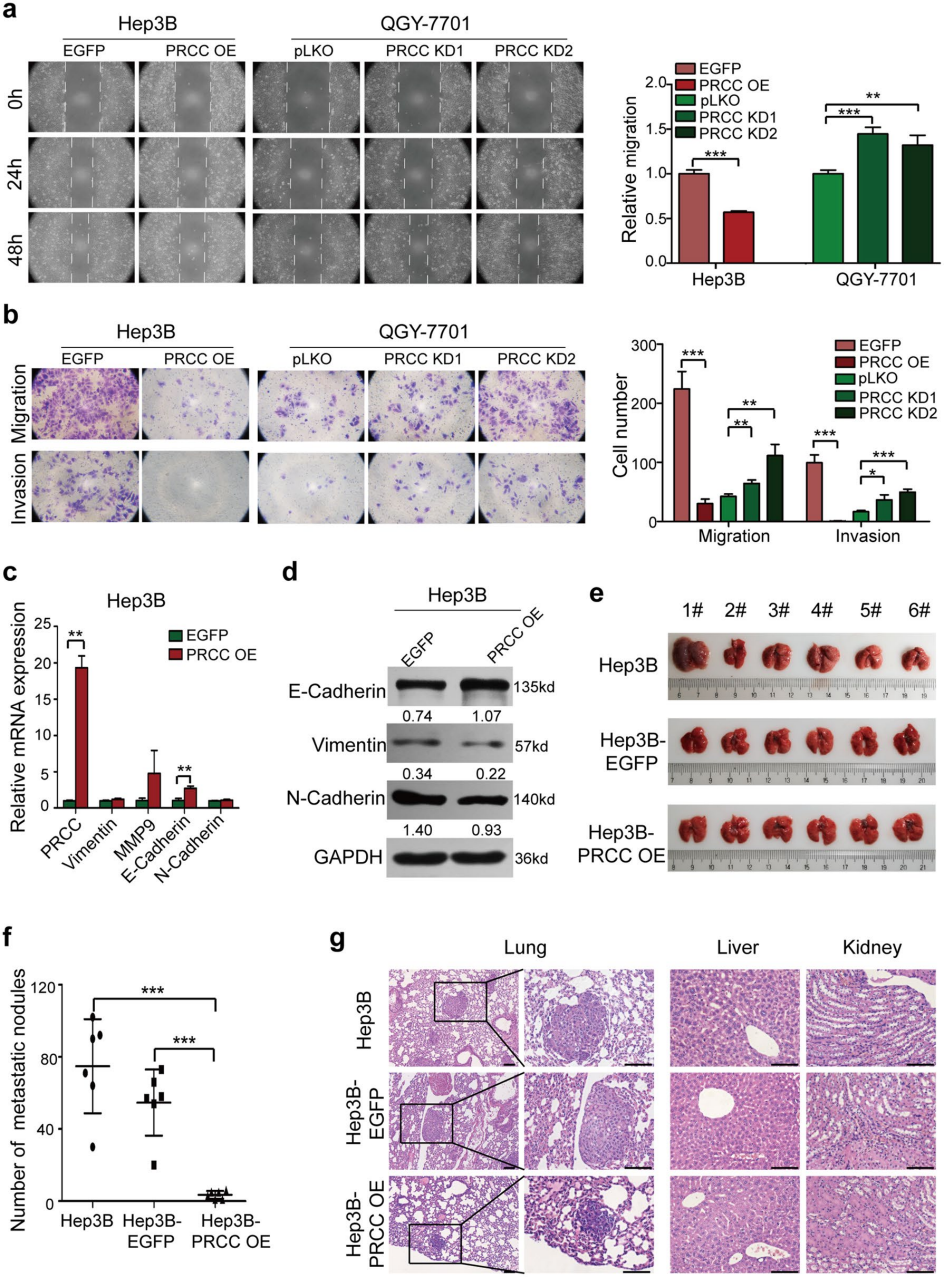
PRCC inhibited HCC cell metastasis in vitro and in vivo. **a** Cell wound and healing assay and the quantitative analysis results (*, *P* < 0.05; **, *P* < 0.01; ***, *P* < 0.001). **b** Transwell assay and the quantitative analysis results (*, *P* < 0.05; **, *P* < 0.01; ***, *P* < 0.001). **c** Expression of the several metastasis-related genes examined by RT-qPCR (**, *P* < 0.01). **d** Expression of the metastasis-related proteins examined by Western blot. The numbers below the bands were the ratios of the gray values of each indicator to that of GAPDH, respectively. **e** Metastatic tumor nodules in lungs of three groups of nude mice. **f** The numbers of metastatic tumor nodules in lungs of three groups (***, *P* < 0.001). **g** The metastatic tumor nodules in lungs, livers and kidneys of nude mice by HE staining. Bar = 100 μm

The overexpression of PRCC significantly inhibited cell migration and invasion of Hep3B and Huh7 cells, while the knockdown of *PRCC* in QGY-7701 and LM3 cells exhibited the opposite results (Fig. 4a, b and Additional file 1: Fig. S2c, d). The transcription of *E-cadherin* was significantly up-regulated (Fig. 4c). Western blot exhibited consistent results (Fig. 4d). A nude mouse model was used by cell injection through tail vein to verify the effect of PRCC on metastasis and tumorigenicity of HCC cells, and the results showed no significant difference in mouse body weight among the Hep3B, Hep3B-EGFP and Hep3B-PRCC OE groups, but the size and numbers of tumors in mouse lung tissues in the Hep3B group and Hep3B-EGFP group were significantly higher than that in the Hep3B-PRCC OE group, and the difference was statistically significant (*P* < 0.05) (Fig. 4e–g). The liver and kidney tissues in the three groups were not found to have metastases (Fig. 4g). The above results indicated that the high expression of PRCC could inhibit the metastasis and tumor formation of HCC cells in vitro and in vivo.

### High expression of PRCC makes cancer cells insensitive to DNA damage

**Fig. 5:**
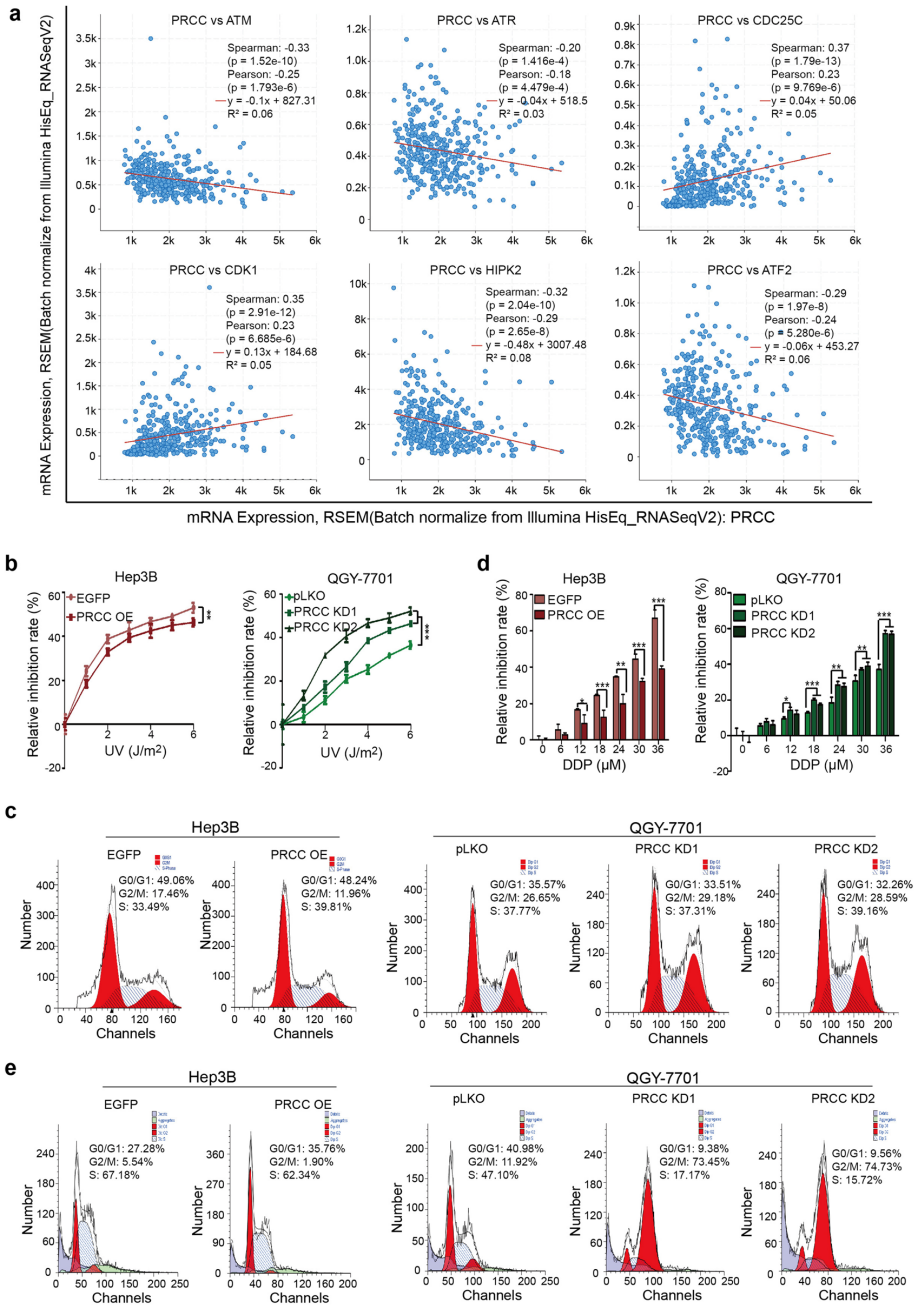
High expression of PRCC made cancer cells insensitive to DNA damage. **a** Co-expression analysis of PRCC and molecules involved in DNA damage repair (http://www.cbioportal.org/results/coexpression). **b** CCK8 assay was used to detect the effect of PRCC on the UV irradiation-treated HCC cells (**, *P* < 0.01; ***, *P* < 0.001). **c** Flow cytometry analysis was used to analyze the cell cycle distribution after UV (5 J/m^2^) treatment. **d** CCK8 assay was used to detect the inhibition rate of DDP on HCC cells with different PRCC expression levels (*, *P* < 0.05; **, *P* < 0.01; ***, *P* < 0.001). **e** Flow cytometry analysis was used to analyze the cell cycle distribution after DDP treatment. All data were representative of three independent experiments

Clinical data analysis showed that PRCC played a "bad" role in the occurrence and development of HCC (Fig. 1). However, PRCC did not show any effect of increasing the malignancy of HCC cells in cytology experiments, but instead inhibited the metastasis and spheroid formation of HCC cells (Figs. 3 and 4). The analysis of co-expression data from TCGA database (http://www.cbioportal.org/results/coexpression) indicated that the expression of PRCC was negatively correlated with the key factors involved in the DNA damage repair process, such as ATM/ATR and HIPK2, and was positively correlated with CDC25C and CDK1 (Fig. 5a) [36, 37]. It could be speculated that PRCC may promote the occurrence and poor prognosis of HCC by affecting DNA damage repair pathways.

Therefore, UV irradiation was used to treat Hep3B and QGY-7701 cells to make DNA damage. The results showed that the proliferation of HCC cells was significantly inhibited by UV irradiation, and the inhibition rate was dose-dependent. The inhibition rate of UV irradiation in PRCC overexpression cells was significantly lower than that in the control group (Fig. 5b). After UV irradiation, the cell cycle distribution in the PRCC overexpression cells changed significantly, the cell ratio in G2/M phase decreased and the cell ratio in S phase increased compared with the control group (Fig. 5c). In addition, the sensitivity of the PRCC overexpression cells to the DNA damage chemotherapeutic drug DDP was significantly reduced (Fig. 5d). After treatment with 30 μM of DDP for 24 h, the cell cycle distribution in the PRCC overexpression cells also changed compared with the control group, the cell ratio in G2/M phase was also decreased (Fig. 5e). The results showed that once DNA was damaged, HCC cells with high protein level of PRCC could not respond as the way normal cells did, they could neither be induced directly into the apoptotic process (indicated by the reduced inhibition rate), nor block the cell cycle to complete DNA repair. Instead, the cells with damaged DNA continued to proliferate (indicated by cell cycle distribution changes) and passed the mutated DNA to daughter cells. The results indicated that the high expression of PRCC affected the response and reduced the sensitivity of cells to DNA damage, and indirectly promoted the progression of HCC.

### PRCC reduces the sensitivity of HCC cells to DNA damage by inhibiting the JNK/ATM/ATR/ATF2 axis

**Fig. 6:**
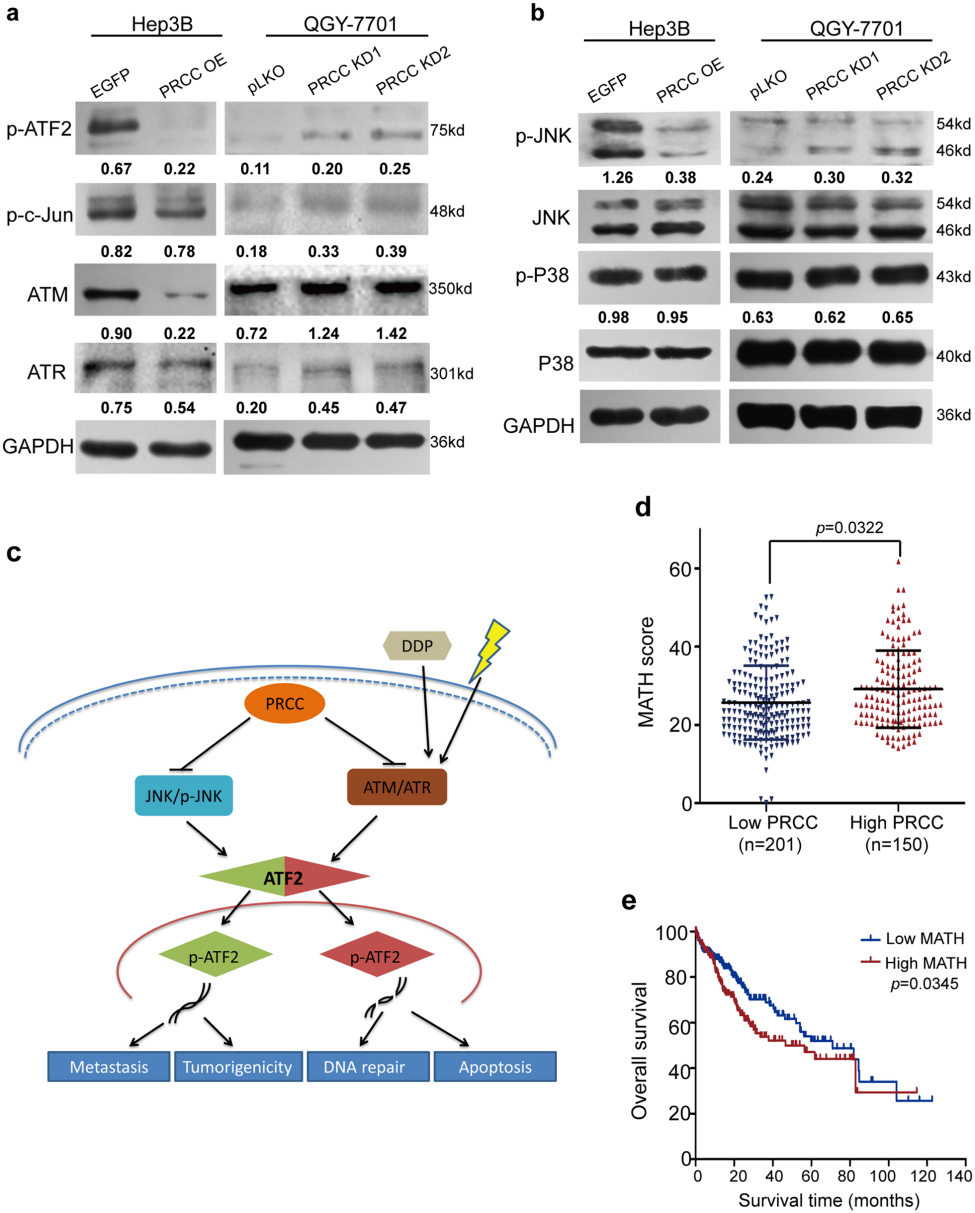
PRCC reduced the sensitivity of HCC cells to DNA damage and led to high ITH and short OS by inhibiting the JNK/ATM/ATR/ATF2 axis. **a** Expression of the DNA damage and repair related proteins examined by Western blot. The numbers below the bands were the ratios of the gray values of each indicator to that of GAPDH. **b** Expression of p-JNK/JNK and p-P38/P38 by Western blot. The numbers below the bands were the ratios of the gray values of p-JNK to that of JNK, and the gray values of p-P38 to that of P38. **c** Schematic diagram of the mechanism of PRCC in HCC cells. **d** The patients with abnormally high expression of PRCC had high MATH (or ITH) (*P* < 0.05). **e** The results of survival analysis showed that high MATH (or ITH) corresponded to short overall survival (*P* < 0.05)

The analysis of TCGA co-expression data showed that *PRCC* expression was negatively correlated with *ATF2* expression (Fig. 5a). ATF2 is a phosphorylation-dependent transcription factor and a substrate of JNK and P38 [11–14]. JNK signaling pathway can be activated by cytokines, growth factors, stress and other factors. The overexpression of PRCC resulted in a significant down-regulation of p-JNK, and did not change p-P38 expression, while the protein level of p-ATF2 was decreased. Knockdown of PRCC expression led to activation of the JNK pathway, which promoted up-regulation of p-ATF2 (Fig. 6a, b). According to reports, ATF2 not only promotes tumor cell metastasis and maintains the characteristics of tumor stem cells through the JNK/ATF2 signaling pathway [15, 16], but also serves as an early response gene to stress and DNA damage [17–19]. ATF2 is a downstream factor of JNK and ATM/ATR signaling pathways, and performs cell response to external stimuli [17]. Co-expression analysis showed that *PRCC* and *ATM/ATR* were negatively correlated (Fig. 5a). Western blot results also verified the same correlation (Fig. 6a).

Based on the above results, the molecular mechanism model of PRCC was deduced (Fig. 6c). In the absence of UV irradiation, the highly expressed PRCC inhibits the activation of ATF2 by inhibiting the JNK signaling pathway, therefore, the colony forming and metastasis abilities of HCC cells are inhibited. For normal cells, once cellular genomic DNA is damaged by UV, radiation, chemotherapeutics or other environmental factors, the JNK and ATM/ATR signaling pathways are activated, then prompting the phosphorylation of ATF2 protein. The activated ATF2 promotes cell apoptosis and DNA damage repair. But when PRCC is highly expressed, the JNK pathway is inhibited, the expression of ATM/ATR is down-regulated, and the expression of p-ATF2 is also down-regulated. At this time, if the genomic DNA of HCC cells is damaged, the HCC cells with high protein level of PRCC cannot respond as normal cells do, they neither directly enter the apoptotic process, nor stop cell cycle to complete DNA repairs. But they carry damaged DNA and mutations to continuously proliferate (Fig. 5c). This not only causes HCC cells to be resistant to radiotherapy and chemotherapeutics, but also causes HCC cells with numerous DNA mutations to become increasingly heterogeneous, which is extremely unfavorable for prognosis of patients.

### High ectopic expression of PRCC leads to high ITH and short overall survival

MATH, generated from whole-exome sequencing (WES) of tumors and their matched normal DNA, was verified to be a simple, quantitative, and universally applicable method for the assessment of ITH [20]. In order to prove the correlation between PRCC and ITH, as well as the relationship between ITH and overall survival of patients, SNV files and clinical data of 365 patients with liver cancer were downloaded from the TCGA database. The sample data of 14 cases were incomplete, and the sample data of 351 cases were actually analyzed. MATH was calculated through R language. The higher the MATH value, the higher the ITH. The patients with high expression of PRCC had higher MATH (or ITH), and the difference was statistically significant (*P* < 0.05) (Fig. 6d). The median value of MATH was used to distinguish the level of ITH. The survival analysis showed that high MATH (or ITH) corresponded to short overall survival (Fig. 6e). It could be concluded that the abnormally high expression of PRCC led to increased intratumoral heterogeneity in HCC patients, which in turn led to a shorter overall survival period for HCC patients.

## Discussion

The processes of tumor occurrence and development are always accompanied by specific changes in the genome, proteome, metabolome and epigenetics [21–23]. In recent years, although researchers have continued to reveal significant changes in molecules and their functions in a large number of cancers, there are still many unclear mechanisms for cancer metastasis, maintenance of cancer stem cells, and resistance to radiotherapy and chemotherapy. There is also a lack of effective diagnostic indicators, especially non-invasive diagnosis based on blood testing in clinic [24]. This study investigated the role and mechanism of PRCC in HCC, for the purpose of elucidating its molecular function, regulatory mechanism and diagnostic value in HCC. The results showed that PRCC has high application potential as a substitute or adjuvant of AFP for clinical diagnosis and prognosis prediction of HCC patients.

However, molecular mechanism study found that PRCC overexpression could inhibit the metastasis and spheroidization of HCC cells. This phenomenon was verified in nude mice. Seemingly, it was inconsistent with the conclusion that the high expression of PRCC was related to poor prognosis of HCC. Through analysis of co-expression data in TCGA database, it was found that the expression of PRCC was significantly negatively correlated with the key factors involved in DNA damage repair process, such as ATM/ATR and HIPK2, and was significantly positively correlated with the G2/M cell cycle factors CDC25C and CDK1 (Fig. 6a). It was speculated that maybe PRCC indirectly leads to poor prognosis of HCC by affecting DNA damage repair pathways and cell cycle regulation. Our results bear out this conjecture.

As we know, in the classic signaling pathways of DNA damage repair and G2/M checkpoint, once cells are exposed to radiation or ultraviolet irradiation, cellular DNA will be broken and cross-linked, which will cause HIPK2, DNA-PK and ATM/ATR to activate and produce a series of responses [25–27]. On the one hand, cell apoptosis is induced through the HIPK2/P53 and ATM/ATR/P53 axis. On the other hand, the ATM/ATR/CHK1/CDC25/CDC2 axis blocks cells from G2 phase to M phase and completes DNA repair, or induces P53-independent cell apoptosis [28–30]. When PRCC is highly expressed, the expression of HIPK2, ATM and ATR proteins is down-regulated, and the expression of CDC25, PLK1, CDK7, etc. is up-regulated. At this time, the radiation- or ultraviolet radiation-induced DNA damage cannot initiate the downstream cell cycle arrest, DNA repair process or cell apoptosis through above molecules. While the increase of CDC25 and other proteins promotes the cell cycle process.

There are many factors that induce DNA mutations in the natural environment, such as ultraviolet radiation, ionizing radiation, some organic compounds, heavy metals, aspergillus flavus, various viruses, etc. [31–34]. The ability of cells to avoid carcinogenesis mainly depends on a sound DNA damage repair system [35]. According to the results we obtained, the high expression of PRCC destroys the sensitivity of the repair system. Therefore, for HCC patients with high expression of PRCC, if radiotherapy or chemotherapy is used and induces DNA damage, it is likely that the treatment effect is not as good as that of patients with low expression of PRCC. Even if the patient does not receive radiotherapy and chemotherapy to induce DNA damage, the microenvironment containing viruses, inflammation factors, reactive oxygen species and aspergillus flavus, etc. will continue to induce DNA damage. The HCC patients with high PRCC expression are not sensitive to DNA damage. As aforementioned, cells carrying DNA damages and mutations will continue to proliferate and lead to an increased heterogeneity in HCC tissues.

Tumor heterogeneity possesses a huge challenge to biomarker prediction and treatment selection. This also means that the higher the heterogeneity of malignant tumors, the worse the prognosis of patients. Our study found that the high expression of PRCC enhances ITH and genomic instability. Although PRCC plays a certain role in inhibiting HCC cell metastasis, its high expression leads to the development and poor prognosis of HCC as a whole. The conclusion clearly explains the role of PRCC in the occurrence and development of HCC for the first time, and provides new clues for HCC diagnosis, treatment guidance, prognosis prediction and targeted therapy.

## Conclusions

PRCC has high application potential as a substitute or adjuvant of AFP for clinical diagnosis of HCC. The high ectopic expression of PRCC not only causes HCC cells to resist to cell death induced by DNA damage, but also causes cancer cells with numerous DNA mutations to become increasingly heterogeneous, finally leading to a poor prognosis for HCC patients. These data suggested PRCC could be a promising therapeutic target in patients with HCC.

## Supplementary Information

**Additional file 1: Figure S1.** High expression of PRCC is associated with poor prognosis of HCC patients in TCGA database (http://www.cbioportal.org/). **a.** The high expression of PRCC is significantly negatively correlated with the overall survival of HCC patients (*P* < 0.05). **b.** The high expression of PRCC is not significantly correlated with the progression-free survival of HCC patients (*P* = 0.183). **Figure S2.** The effects of PRCC on the biological behavior of HCC cells. **a.** PRCC has no significant effect on the proliferation of HCC cells. **b.** PRCC inhibits the colony forming ability of HCC cells (*, *P*<0.05). **c, d.** PRCC inhibits the migration and invasion of HCC cells in vitro. The histograms are the quantitative analysis results (*, *P* < 0.05; **, *P* < 0.01; ***, *P* < 0.001). All data were representative of three independent experiments. **Table S1.** Primer sequences of RT-qPCR. **Table S2.** Antibody information.

## Data Availability

The data sets used and/or analyzed during the current study are available from the corresponding author on reasonable request.

## Abbreviations

AFP: Alpha-fetoprotein
DDP: Cisplatin
ELISA: Enzyme linked immunosorbent assay
HCC: Hepatocellular carcinoma
IF: Immunofluorescence
IHC: Immunohistochemistry
ITH: Intratumoral heterogeneity
KD: Knockdown
MATH: Mutant-allele tumor heterogeneity
OE: Overexpression
PRCC: Proline rich mitotic checkpoint control factor
PVTT: Portal vein tumor thrombus
ROC: Receiver operating characteristic curve
RT-qPCR: Quantitative reverse transcription PCR
SNV: Single nucleotide variation
UV: Ultraviolet
WES: Whole-exome sequencing
