# Supplementary material for: PRCC reduces the sensitivity of cancer cells to DNA damage by inhibiting JNK and ATM/ATR pathways and results in a poor prognosis in hepatocellular carcinoma
Source: Cell Biosci. 2021 Oct 29;11:185. doi: 10.1186/s13578-021-00699-x (PMC8555229; doi:10.1186/s13578-021-00699-x)
Supplement: Supplementary file 1 — Additional file 1: Figure S1. High expression of PRCC is associated with poor prognosis of HCC patients in TCGA database (http://www.cbioportal.org/). a. The high expression of PRCC is significantly negatively correlated with the overall survival of HCC patients (P < 0.05). b. The high expression of PRCC is not significantly correlated with the progression-free survival of HCC patients (P = 0.183). Figure S2. The effects of PRCC on the biological behavior of HCC cells. a. PRCC has no significant effect on the proliferation of HCC cells. b. PRCC inhibits the colony forming ability of HCC cells (*, P<0.05). c, d. PRCC inhibits the migration and invasion of HCC cells in vitro. The histograms are the quantitative analysis results (*, P < 0.05; **, P < 0.01; ***, P < 0.001). All data were representative of three independent experiments. Table S1. Primer sequences of RT-qPCR. Table S2. Antibody information. [file 13578_2021_699_MOESM1_ESM.docx]

**Additional information**

**Additional experimental procedures**

**Cell migration and invasion**

About 3×10^4^ cells /200 μl (migration experiment) or 6×10^4^ cells /200 μl (invasion experiment) were added to the upper chambers in transwell. The transwell chambers were pre-coated with matrigel when detecting the invasion ability. After being cultured in an incubator for 48 h, the remaining cells in the chamber were wiped out by cotton swabs, and the migrated or invasive cells were fixed with formaldehyde, stained with crystal violet, and photographed under a microscope.

**MATH calculation**

The MATH calculation program is as follows:

# Read files and calculate MATH values one by one

for (i in 1: length (myFilelist))

{temp <- read. table (myFilelist[i], header=T, sep="\t", fill=TRUE, quote = "")

temp$tumor_vaf <- 100*(a$t_alt_count/a$t_depth)

# Choose mutations with vaf > 7.5

MAF <- temp$tumor_vaf[temp$tumor_vaf > 7.5]

# Calculate MATH

MIDMAF<- median (MAF)

MAD <- mad (MAF, constant=1.4826, na.rm = TRUE, low = FALSE, high = FALSE)

MATH <- 100*MAD/MIDMAF

# Store results

result[i,2] <- MATH

result[i,1] <- substr (myFilelist[i],1,12)

result[i,3] <- nrow (temp)

result[i,4] <- length (MAF)}

write.csv (result, "MATHBRCA.csv", row.names = F)

**Additional figures**

**Figure S1**

**
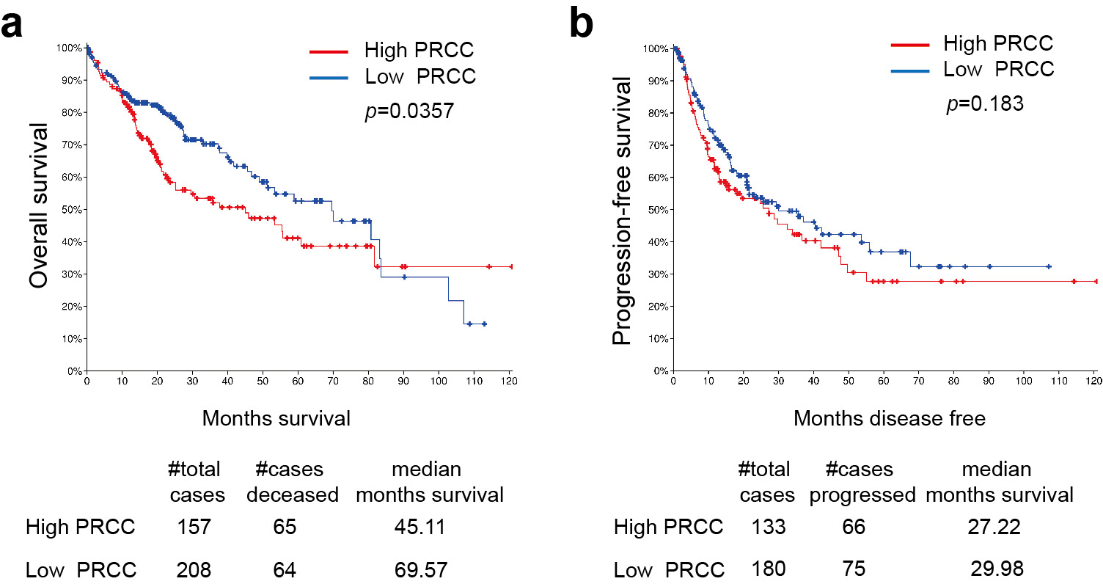
**

**Figure S1.** High expression of PRCC is associated with poor prognosis of HCC patients in TCGA database. **a.** The high expression of PRCC is significantly negatively correlated with the overall survival of HCC patients (*P*<0.05). **b.** The high expression of PRCC is not significantly correlated with the progression-free survival of HCC patients (*P*=0.183).

**Figure S2**


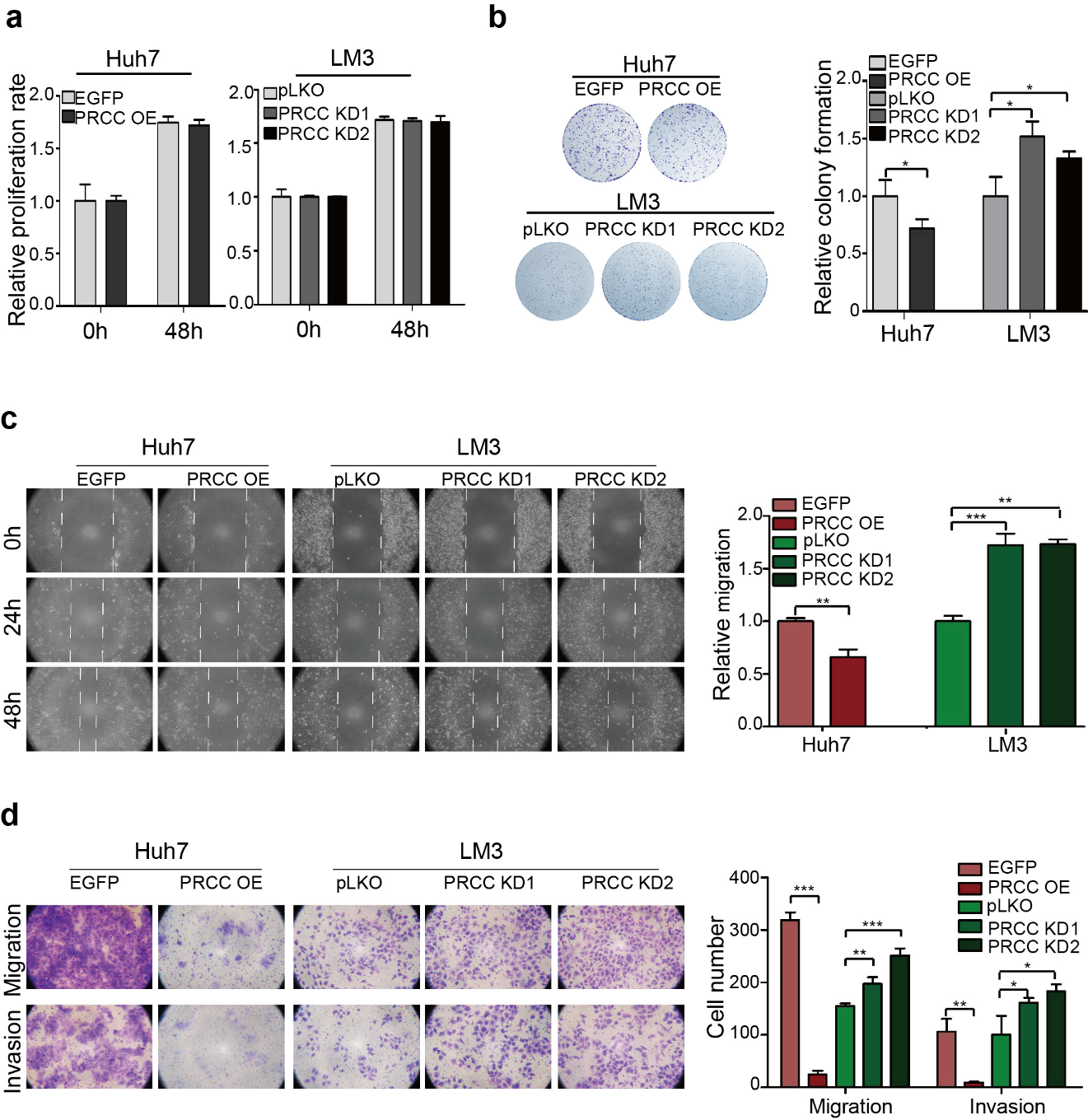


**Figure S2.** The effects of PRCC on the biological behavior of HCC cells. **a.** PRCC has no significant effect on the proliferation of HCC cells. **b.** PRCC inhibits the colony forming ability of HCC cells (*, *P*<0.05). **c, d.** PRCC inhibits the migration and invasion of HCC cells *in vitro*. The histograms are the quantitative analysis results (*, *P*<0.05; **, *P*<0.01; ***, *P*<0.001). All data were representative of three independent experiments.

**Table S1.** Primer sequences of RT-qPCR

| Table S1 Primer sequences of RT-qPCR | | |
| --- | --- | --- |
| Gene |  | Sequence (5' -> 3') |
| *PRCC* | F: | CCTGGGGACGACTACAGCTA |
|  | R: | GCAGCCGCTTAAATGCTTCG |
| *Vimentin* | F: | GACGCCATCAACACCGAGTT |
|  | R: | CTTTGTCGTTGGTTAGCTGGT |
| *MMP9* | F: | TCTATGGTCCTCGCCCTGAA |
|  | R: | CATCGTCCACCGGACTCAAA |
| *E-Cadherin* | F: | AAAGGCCCATTTCCTAAAAACCT |
|  | R: | TGCGTTCTCTATCCAGAGGCT |
| *N-Cadherin* | F: | AGCCAACCTTAACTGAGGAGT |
|  | R: | GGCAAGTTGATTGGAGGGATG |
| *GAPDH* | F: | ACAACTTTGGTATCGTGGAAGG |
|  | R: | GCCATCACGCCACAGTTTC |

**Table S2.** Antibody information

| Table S2. Antibody information | |
| --- | --- |
| IHC | anti-PRCC antibody (HPA019463, Sigma, USA. Dilution 1:50) |
| IF | anti-GFP antibody (ab1218, Abcam, USA. Dilution 1:500) |
|  | anti-EpCAM antibody (2929, CST, USA. Dilution 1:100) |
|  | anti-β-Catenin antibody (8480, CST, USA. Dilution 1:100) |
|  | anti-CD44 antibody (A12410, ABclonal, CN. Dilution 1:100) |
|  | anti-CD133 antibody (A12711, ABclonal, CN. Dilution 1:100) |
| WB | anti-PRCC antibody (HPA019463, Sigma, USA. Dilution 1:200) |
|  | anti-E-Cadherin antibody (21473, SAB, USA. Dilution 1:1000) |
|  | anti-Vimentin antibody (A11952, ABclonal, CN. Dilution 1:800) |
|  | anti-N-Cadherin antibody (A0433, ABclonal, CN. Dilution 1:800) |
|  | anti-p-ATF2 antibody (AP0128, ABclonal, CN. Dilution 1:800) |
|  | anti-ATM antibody (A19650, ABclonal, CN. Dilution 1:1000) |
|  | anti-ATR antibody (19787-1-AP, Proteintech, CN. Dilution 1:500) |
|  | anti-p-c-JUN antibody (9164, CST, USA. Dilution 1:1000) |
|  | anti-p-JNK antibody (4668, CST, USA. Dilution 1: 1000) |
|  | anti-JNK antibody (9252, CST, USA. Dilution 1:1000) |
|  | anti-p-P38 antibody (4511, CST, USA. Dilution 1:1000) |
|  | anti-P38 antibody (9212, CST, USA. Dilution 1:1000) |
|  | anti-GAPDH antibody (KC5G5, Aksomics, CN. Dilution 1:5000) |
